# Piroxicam Adsorption over Graphene OXIDE Nanosheets: A DFT-ONIOM Study

**DOI:** 10.1021/acsomega.5c13355

**Published:** 2026-05-06

**Authors:** Alejandro M. Velázquez-García, Sandy M. Pacheco-Ortin, Benjamín Velasco-Bejarano, Roberto Mejía-Olvera, Esther AgacinoValdés

**Affiliations:** † Centro de Investigaciones Teóricas, Facultad de Estudios Superiores Cuautitlán, Universidad Nacional Autónoma de México, Cuautitlán Izcalli, Edo. México CP 54740, Mexico; ‡ Departamento de Química, Facultad de Estudios Superiores Cuautitlán, Universidad Nacional Autónoma de México, Cuautitlán Izcalli, Estado de México CP 54740, Mexico

## Abstract

A theoretical study of piroxicam (PIR) adsorption over graphene (G) and graphene oxide (GO) sheets was made to find out whether these nanomaterials could be integrated as an additional element into the existing hybrid methodologies of water treatment, to eliminate this contaminating drug. The G-sheet was modeled starting from a polycircumcoronene cluster of 78 carbon atoms, which facilitated the good accommodation of the PIR drug over the sheet; to minimize edge effects, a chemical environment of carbons and hydrogens (C_72_H_30_) was added around the initial structure, using the ONIOM methodology, to thus obtain a final cluster of C_150_H_30_; after this, the G cluster was functionalized to obtain the GO-sheet with a C/O ratio of 5.33. The calculations were performed using the Density Functional Theory formalism implemented in Gaussian09 package. The PIR adsorption energy was about 19–21 kcal mol^–1^, with adsorption distances less than 3.00 Å and a significant change of 327 meV in the gap (HOMO–LUMO) of the sheet, after the adsorption process. Some elongations of the bonds in the adsorbed PIR with respect to its free form were also observed. All these results indicated a favored chemisorption process and allowed us to confirm that GO-sheets could be a good adsorbent for this drug. The G-sheet also showed a good behavior as an adsorbent material.

## Graphical abstract

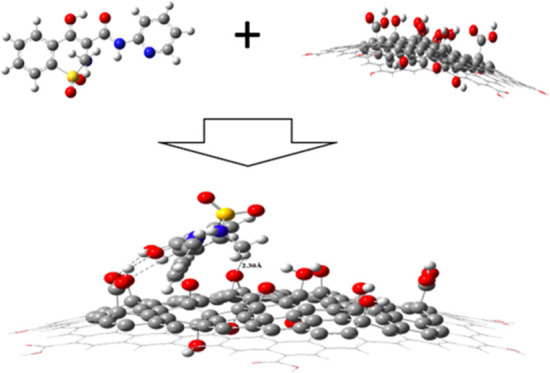

## 1 Introduction

In recent decades, diverse pharmaceuticals, dyes, fertilizers, pesticides, and other personal care and cosmetic products have emerged as a new class of environmental pollutants called emerging contaminants (ECs), with harmful effects on both human health and natural flora and fauna.^1,2^ ECs are compounds of diverse origin and chemical nature present not only in wastewater but also in groundwater, domestic water, and agricultural water. Since these EC are present at low concentrations, they had long gone unnoticed. However, their effects are already becoming noticeable, as their concentrations in water are beginning to exceed experimental detection critics’ limits, raising concerns about the environmental risks they are causing.^3^

Particularly, nonsteroidal anti-inflammatory drugs (NSAIDs) are a group of substances with significant anti-inflammatory, antipyretic, and analgesic properties, and they constitute one of the most widely prescribed therapeutic groups worldwide. But they are also a group of medications that are eliminated from the body in a high percentage,^4^ and for this reason they are classified as EC. Piroxicam (PIR) is one of these drugs that, although very useful for relieving inflammation and pain, has many adverse effects. Therefore, its presence in various water sources represents a risk and exemplifies the urgent need to implement degradation methodologies to eliminate it due to its potential impact on ecosystems, water quality, and environmental health.

Existing methodologies for degrading ECs are often referred to as “hybrid” because they combine techniques such as advanced oxidation, the use of sunlight or UV light, reverse osmosis, and sedimentation, among others; however, the inclusion of techniques based on adsorption processes with the use of adsorbent surfaces is still scarce, though it is not an expensive technology and integrates well in the wastewater treatment plants,^1^ particularly when the pollutants are pharmaceuticals.^5^

Among the adsorbent surfaces under investigation is graphene, which has attracted much attention due to its very special structure and physicochemical properties, which have generated important applications in biomedicine, energy, and water treatment.^6^ Recently, various graphene-based materials have played a very important role in water treatment; however, although the literature is scarce, it is already recognized that graphene-based materials can be used in water treatment as adsorbent surfaces^7^ and that their surfaces can be modified to improve not only their adsorbent but also their electrical, electronic, mechanical, optical, and thermal properties.^6^

G-sheets can also improve their adsorbent properties when they are functionalized, since their covalent and noncovalent interactions with adsorbates are strengthened. Looking for new graphene-like materials with high adsorption capacities for organic pollutants and high dispersion properties in aqueous solutions, Zhao et al. in 2011,^7^ tested the graphene functionalized by sulfonic acid groups; the adsorption of naphthalene and 1-naphthol molecules over sulfonated graphene was studied in both experimental and theoretical ways. The results showed that the adsorption percentages of these molecules increased as the sulfonated graphene content in an aqueous solution increased; the naphthalene molecule was adsorbed parallel to the sulfonated graphene sheet in a π-sandwich noncovalent interaction with adsorption distances of 3.57 Å and 3.71 Å and an adsorption energy of 1.96 and 2.01 kcal mol^–1^, respectively. 1-Naphthol was adsorbed in a tilted position, favoring a noncovalent interaction through the –OH group at adsorption distance of 2.58 Å and released energy of 3.26 kcal mol^–1^. This investigation showed that organic pollutants with aromatic rings and heteroatoms could be adsorbed by functionalizing graphene sheets with groups containing heteroatoms, and these sheets could be good adsorbents in aqueous solutions.

In this way, another of the functionalized variants of graphene that is being most investigated is GO sheets, which contain various functional groups distributed over its surface, such as carboxyl, carbonyl, epoxide, and hydroxyl, which give it significant reactivity and a great capacity to adsorb molecules; this, together with the fact that it can be an even more stable material than graphene itself, makes it a successful material.^8^ Because of the unique chemical structure and adsorbent properties of G-sheets and GO-sheets, many theoretical and experimental studies have been carried out on their application in water treatment. In 2014, Zhao et al.,^9^ using density functional theory (DFT) formalism and the M06–2X functional, demonstrated that the adsorption of HCN over reduced GO was stronger than over G; in this process, the formation of hydrogen bonds mediated by carboxyl, hydroxyl, and epoxide groups was found. In another study published in 2011, Tang and Cao^10^ used DFT in a periodic scheme to investigate the adsorption of nitrogen oxides (NO_*x*_ for *x* = 1, 2, and 3) over G and GO; they concluded that, in total agreement with experimental results, the adsorption over GO turned out to be stronger (chemical adsorption) than over G due to (i) the presence of the –OH and –C=O groups, which increased the charge transfer from the nitrogen of NO_*x*_ to GO, and (ii) the formation of −O–H···O (N) hydrogen bonds and weak C···N and C···O covalent bonds. In a 2017 experimental work, Zhu et al.^11^ demonstrated the ability of GO sheets to remove the metformin, a drug used in the treatment of diabetes, from water; they found that this process depended on temperature, pH, and ionic strength media. Besides, the adsorption kinetic experiments revealed that almost 80% of the removal of metformin was achieved. Summing up, the adsorption of this drug to GO was achieved within 20 min in a thermodynamically spontaneous and exothermic process with a maximum adsorption at a pH between 6 and 8.

In aquatic complex matrices like the wastewater, GO has been used for the removal of various ECs, including diverse pharmaceutical drugs; this versatility of GO would not cause interference between them because first, it exhibits a large surface area, which provides more binding sites for diverse contaminants, and second, it exhibits a high adsorption capacity, which makes it possible for GO to interact and adsorb a wide range of ECs without losing its efficiency as a versatile adsorbent material. On the other hand, GO can be easily functionalized to selectively target specific contaminants.^12,13^

Ersan et al.^14^ have reported that between 2012 and 2015 more than 40 articles had already been published studying the adsorption of ECs on G and GO sheets, which had demonstrated the attention these nanomaterials were receiving; they concluded, in agreement with Zhu et al., that not only are the characteristics and properties of the adsorbent material important but also the properties of the solution could have influence.^11^ Finally, it has also been suggested that it is necessary to study the various ECs separately to better understand their adsorption process.

The applications of graphene and graphene-based materials for the improvement of the environment have advanced greatly; however, due to the constraints of cost, stability, and preparation techniques, they are still at the stage of laboratory research, but it is expected that soon the use of these materials will increase in fields such as water pollution treatment and drinking water purification, among others.^15^

Precisely, the aim of this work is to evaluate, through a theoretical study, the potential possibilities of graphene and graphene oxide as adsorbent materials of piroxicam, an NSAID-type drug considered within the group of the emerging water pollutants; if so, these nanomaterials could help degrade these contaminants.

## 2 Computational Methods

### 2.1 Computational Details

The calculations were made using the combination M06–2X/6–31 G** of density functional and basis set, included in the Gaussian 09 package.^16^ This functional includes a Hartree–Fock exchange term to improve the description of noncovalent interactions, so it is parametrized for systems in which this kind of interactions play an important role. Because of this, it is very useful for studying adsorption phenomena, as in this work.^9,17,18^ In addition, the 6–31 G** base set is suitable for large molecular systems^19^ and combined with the M06–2X functional can provide reliable and accurate results with a lowest computational cost;^20,21^ therefore, this combination is suitable for the study of adsorption complexes.

The geometries of all the chemical species in study were optimized, first in gas phase and then considering the solvent effect, through a solvent model density scheme.^22^ This model is convenient because it has been used successfully in DFT calculations combining the 6–31G* base with functionals from the same series as the M06–2X.^18^ In both optimization procedures (gas phase and solvent), the corresponding frequency analysis was made to confirm that the stationary points were truly minima in the PES. Finally, it is important to note that solvent effect was necessary for providing a more realistic representation of the problem in study, that is, the presence of pollutants in water.

### 2.2 Construction of G and GO Models

GO is a material very sensitive to the synthesis ways used and the temperature, so we can see it as a dynamic structure that can change its composition easily;^23^ however, within its structural randomness, there are certain regularities that have been found and have allowed us propose some structural models. For example, it is known that its properties depend to the oxygen content, which is related to the functional groups that are present.^24^ One of the most widely used models in the literature is the Lerf–Klinowski model, where the hydroxyl and epoxy groups are located on the basal plane of the graphene sheet and the carboxyl groups are located at the edges,^25,26^ a proposed derived from ^13^C and ^1^H NMR spectra analysis. In agreement with the Lerf-Klinowski model it was also found that the most energetically favorable configuration of GO has been found to contain agglomerates formed by one epoxy for every two hydroxyl groups. Liu et al.,^27^ using a periodic DFT formalism, reported C–O distances of 1.464 Å for epoxy groups; they also found that the presence of these two functional groups in both sides of the sheet (trans conformation) produced a more stable configuration than on one side only. Similarly, theoretical studies by Luo et al.^28^ demonstrated that trans conformation was more thermodynamically stable.

With respect to the oxygen content, Gao et al. found a probable C/O ratio of 2.44 in their XPS and solid-state NMR studies;^29^ on the other hand, Qiao et al. proposed GO models with an overall C/O ratio range of 1.86–18.05; these models were studied by FTIR, XPS, vacancy defects, and curvature properties, and compared with available experimental results; they found that the model with a C/O value about 2.93, among other characteristics, was the best.^24^ However, Lojka et al. prepared and characterized various samples of partially oxidized graphite oxide and evaluated the C/O ratios from XPS, EDS, and EA, with the ranges of 3.1–12, 2.5–12.5, and 3.8–7.3, respectively. These works proved that GO structures are sensitive to the experimental synthesis conditions.^30^ We decided to model the GO considering a C/O ratio within the ranges obtained by Lojka but aiming more toward the lower bound and also to the Qiao and Gao parameters.

With the above background in mind, we began to model our GO nanosheet. First, a graphene nanosheet was modeled starting from the optimized coronene molecule with formula C_12_H_24_, as it is shown in Figure 1a. The coronene molecule was the starting point for modeling, as its structure and properties have been shown to be like graphene.^31–33^ The coronene molecule was then grown to obtain a rectangular polycircumcoronene cluster of 78 carbon atoms, thus facilitating the adsorption of piroxicam, a drug with a length of approximately 13.68 Å, as it is shown in Figure 1b. To minimize edge effects, a chemical environment of carbons and hydrogens (C_72_H_30_) was modeled around this structure, using the ONIOM methodology implemented in Gaussian,^34^ to finally obtain a polycircumcoronene cluster of 150 carbon atoms that simulates the graphene sheet (C_150_H_30_) as Figure 1b shows.

**Figure 1:**
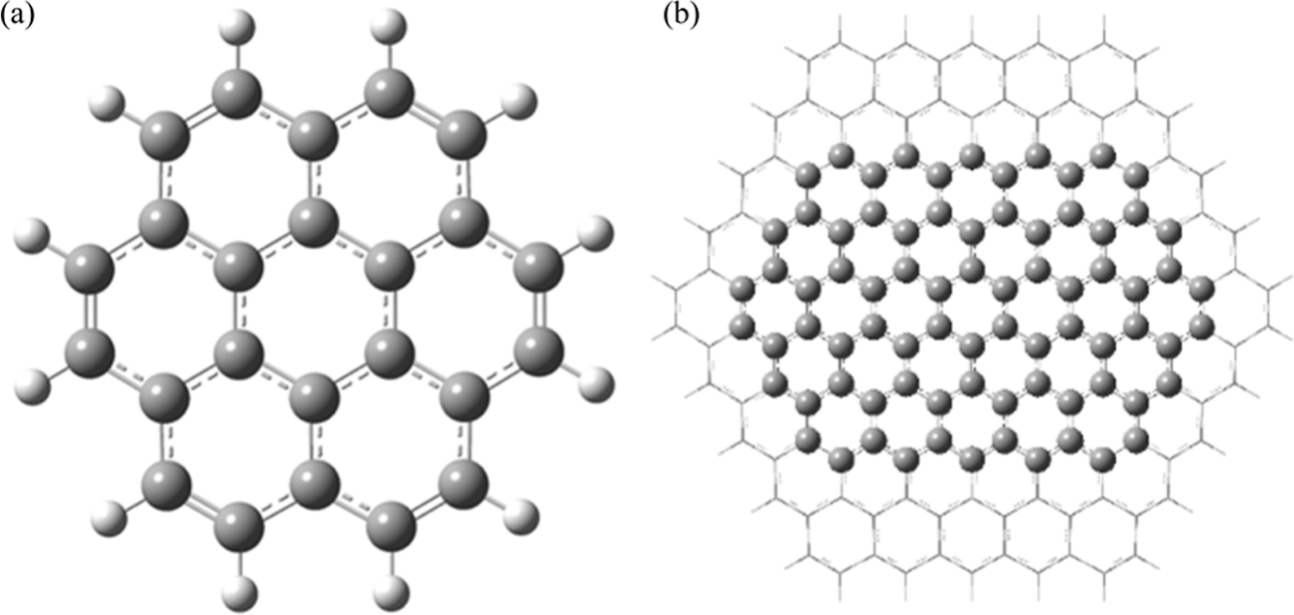
Optimized geometry of: (a) coronene molecule (C_24_H_12_); (b) polycircumcoronene sheet (C_150_H_30_). The partition between the internal part (high) and the external part (low) can be observed.

Starting from this ONIOM-type polycircumcoronene cluster, the GO cluster was subsequently modeled, as explained later. As it is known, the ONIOM methodology was developed by Morokuma et al.^35,36^ and is very convenient for molecular systems with a lot of atoms and electrons.^37,38^

To apply this methodology, the graphene sheet was partitioned into an internal part of 78 carbon atoms (high zone) calculated with a higher level of theory (in this work: M06–2X/6–31G** and an external part of C_72_H_30_ (low zone), with a lower level of theory (in this work: HF/3–21G); optimized geometries and total energies could be obtained with this methodology for all the species in study.

The functionalization of graphene-nanosheet for obtaining the GO-nano sheet considered the Lerf-Klinowski model and the trans conformation according to the results mentioned above.^26,27^ For this geometry, two types of epoxy/OH agglomerates were inserted in the high zone, each consisting of two –OH groups for each epoxy group and one –OH groups for each epoxy group; at the high–low interface, two carboxyl groups and two hydroxyl groups were inserted; at the outermost edge of the low zone, two –OH groups and four carboxyl groups were added; and finally, an epoxy group and two hydroxyl groups were added at the opposite side of the sheet (trans position) in the high zone. This model generated a C/O ratio of 5.33, which was considered to be convenient.

### 2.3 Methodology

The optimizations of the geometries were made first for the free PIR molecule and GO sheet and subsequently for the adsorption complexes PIR-GO. For characterizing the adsorption complexes formed, the adsorption distances, Frontier orbital maps, the molecular electrostatic potential (MEP) maps were obtained. Besides, other adsorption descriptors like adsorption energies (*E*_ads_), the HOMO–LUMO gap (*E*_g_), and the change of the gap during the adsorption process (Δ*E*_g_) were calculated. The corresponding eqs [[Disp-formula eq1]–[Disp-formula eq3]] are presented below

1 $$E_{ads}=\left(E_{PIR}+E_{GO}\right)-E_{adsorp\;complex}$$

2 $$E_{g}=E_{LUMO}-E_{HOMO}$$

3 $${\Delta E}_{g}=E_{g}\left(Adsorp\;Complex\right)-E_{g}\left(GO-free\right)$$

## 3 Results and Discussion

### 3.1 Structure and Reactivity of the Graphene Oxide Sheet

Figure 2a,b shows the optimized surface of the GO-sheet in top and side views, respectively. Note that, unlike the G-sheet, the GO-sheet has lost planarity as the result of the hybridization change (sp^2^ to sp^3^) of the carbons where the epoxide, –OH, carbonyl, and carboxyl groups have been substituted. Besides, Table 1 summarizes some descriptors of GO-sheet modeled like the distances C–O, HOMO–LUMO gap (*E*_g_), and the dipolar moment (μ). The distances of C–O corresponding to the epoxide and –OH substitutions are slightly smaller than Liu et al.’s results, which is understandable considering that the composition of the GO modeled in both works (C/O ratio) was not the same exactly.

**Figure 2:**
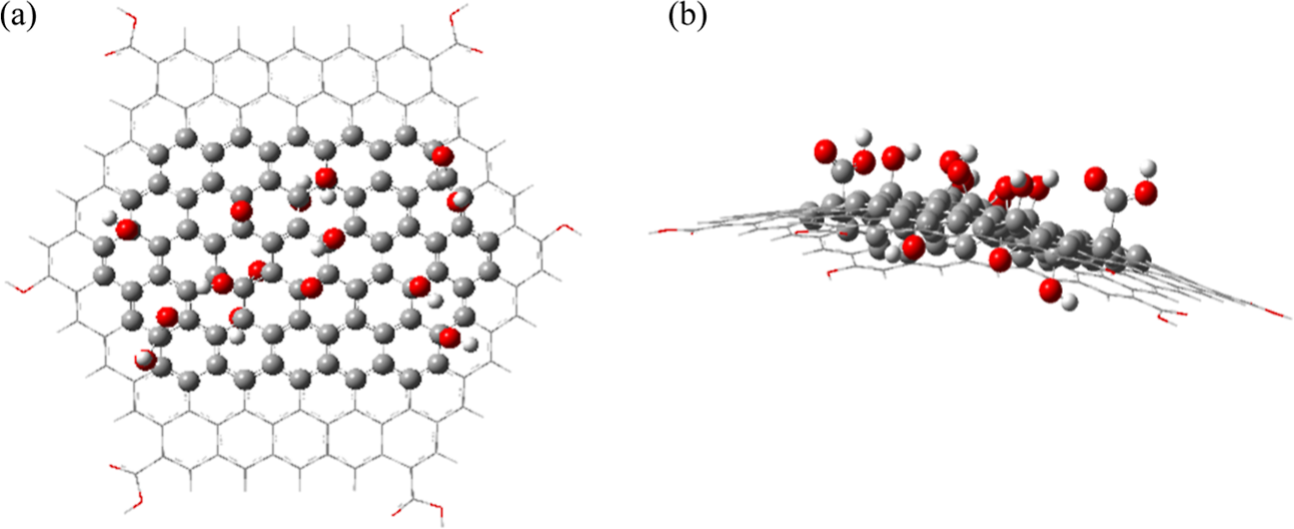
Optimized geometry of the GO sheet: (a) top view; (b) side view.

**Table 1:** Descriptors of the Graphene Oxide Sheet Model^a^

C/O ratio	*d*(C–O)/Å in epoxide	*d*(C–O)/Å in –OH	*E*_g_/eV	μ/D
5.33	1.436, 1.426*	1.446, 1.438*	5.00	20.73

a The distances with an asterisk mark correspond to the groups located on the opposite side of the sheet.

The distances of C–O corresponding to the epoxide and –OH substitutions are slightly smaller than Liu et al.’s results, which is understandable considering that the composition of the GO modeled in both works (C/O ratio) was not the same exactly.

As can be seen in Table 1, the hybridization changes of the carbons bonded to oxygens and the presence of these very electronegative elements have influenced the HOMO–LUMO gap, which increased approximately 1 eV with respect to the G-sheet; the polarity was also increased significantly.

Figure 3 shows a mapping of the Frontier orbitals (HOMO and LUMO) and the MEP map (for a range of ±1.000 × 10^–2^); notice that only the high zone of the ONIOM structure in the sheet of GO has been considered, as it is of the greatest interest. You can notice in Figure 3a,b that available states in both Frontier orbitals correspond to orbitals with a π-symmetry, coming from consecutive carbons with sp^2^ hybridization present. On the other hand, the MEP map in Figure 3c shows the charge gradients, which allow us to explain the polarity of this structure and its reactivity as adsorbent material, as we will see in the next section.

**Figure 3:**
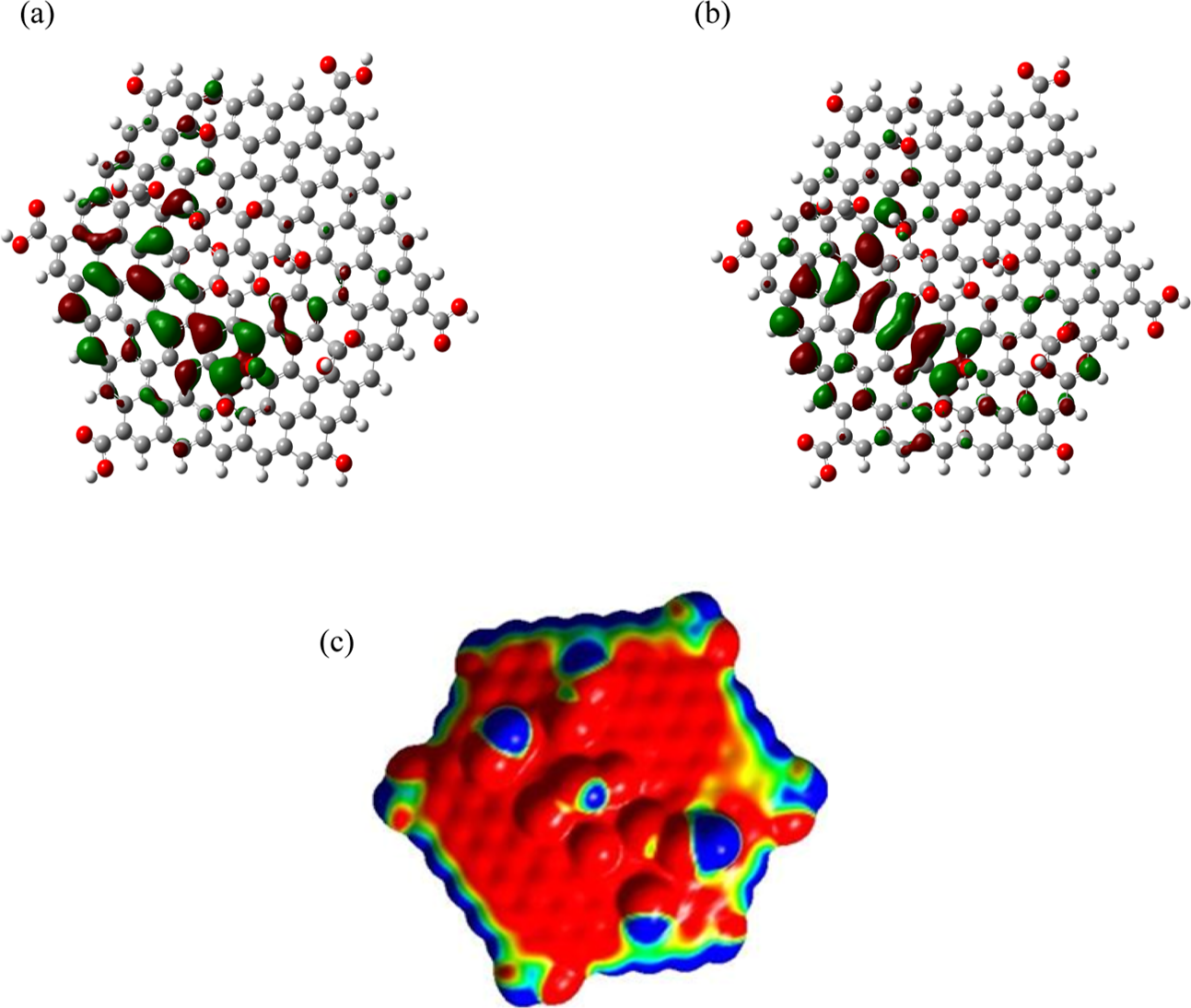
Mapping of Frontier orbitals for the GO sheet: (a) HOMO orbital; (b) LUMO orbital; (c) MEP.

### 3.2 Structure and Reactivity of the Piroxicam Molecule (PIR)

Figure 4 shows the optimized geometry of PIR, which corresponds to the enol-conformer. In this molecule can be observed three fragments: (i) At the center, a benzothiazine ring containing a sulfur atom forming a sulfoxide group, a nitrogen atom bonded to a methyl group, and an –OH group; (ii) a peripheral benzene ring bonded by two positions to the benzothiazide ring to maintain the planarity of the molecule; (iii) a carboxamide group (R–CO–NR′R″), bonded to the benzothiazide ring (R), where R′ is an H atom and R″ is a pyridyl ring. In the conformation presented, the carbonyl group and the –OH are in adjacent positions, forming a hydrogen bond which further reinforces the planarity of the molecule.

**Figure 4:**
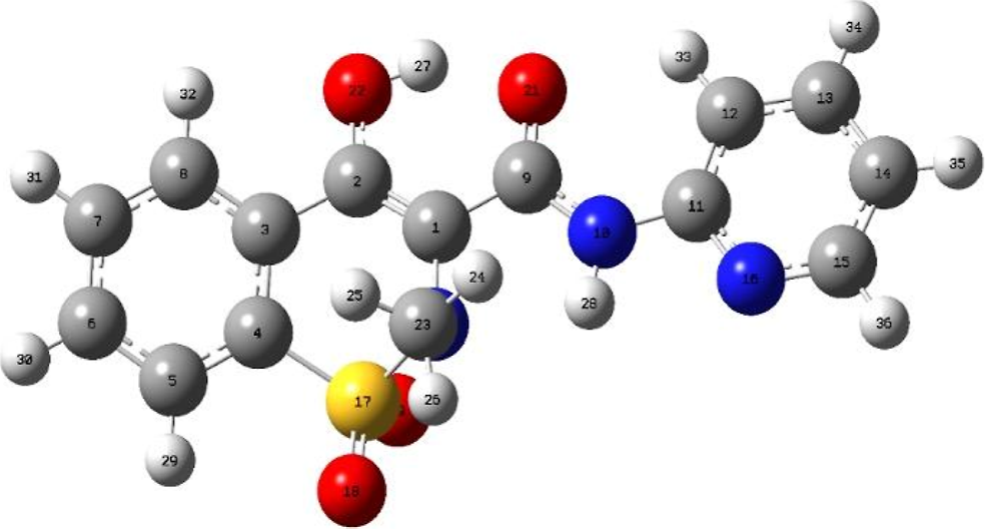
Optimized geometry of the PIR molecule.

The PIR molecule has an electron-withdrawing side (sulfoxide and pyridyl groups) and an electron-donating side (carbonyl and –OH groups); it also has a conjugated system that extends through the molecule and is reinforced toward the ends, resulting in a delocalized electron density of type π and electrostatic effects due to the heteroatoms present in the center of the molecule.

Finally, it is to be expected that this molecule, when adsorbed over the OG sheet, prefers to approach by its oxygens (carbonyl and sulfoxide groups), by the –OH and > N–H groups, or in parallel to the π-system of the sheet. Therefore, the formation of hydrogen bonds and π stacking will be the most important noncovalent interaction.

### 3.3 Study of the PIR-G and PIR-GO Adsorption Complexes

Figure 5 shows comparatively the optimized geometries of the PIR-G and PIR-GO adsorption complexes in Figure 5a,b, respectively.

**Figure 5:**
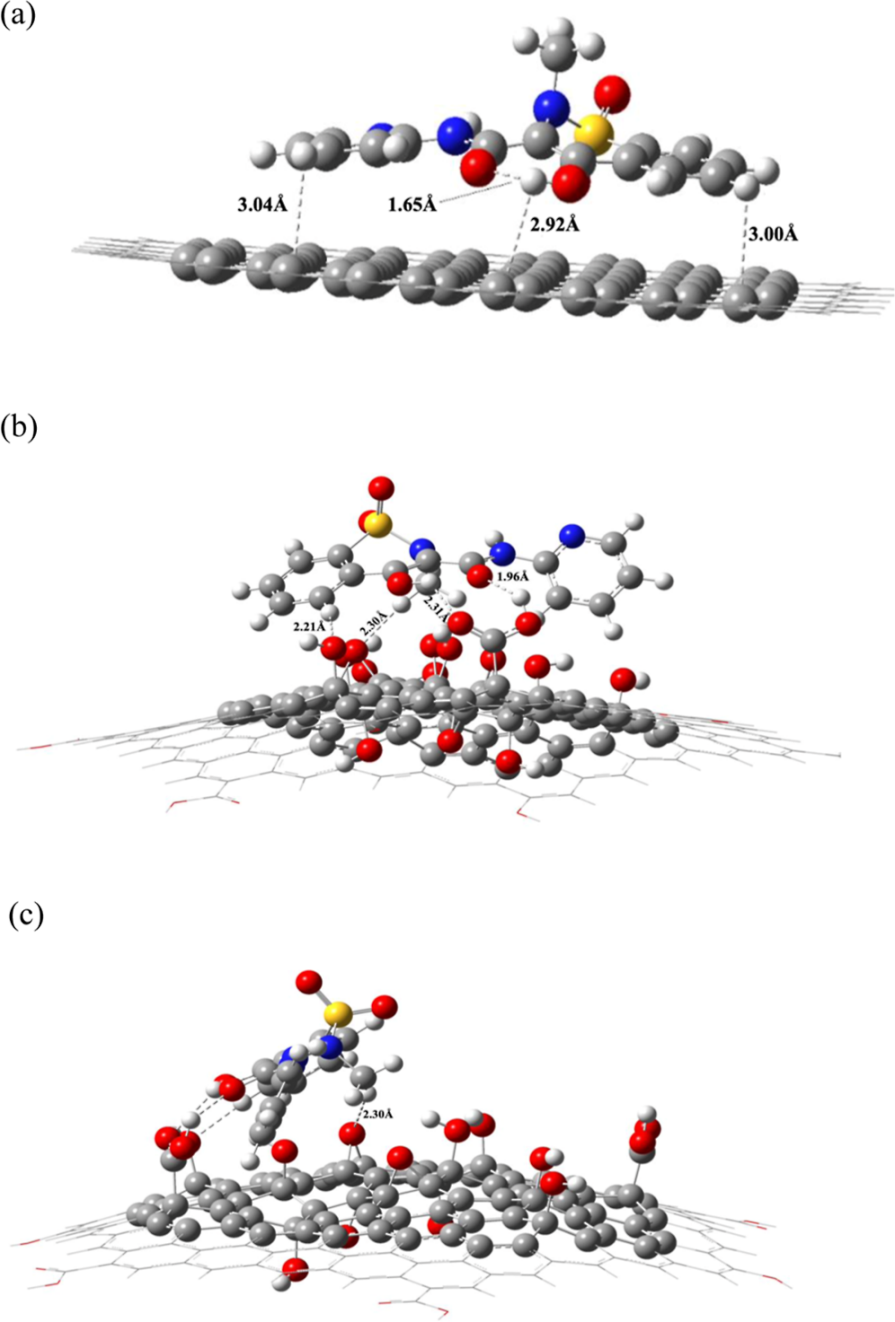
Optimized geometries of the adsorption complexes: (a) PIR-G, with an adsorption energy of 20.809 kcal mol^–1^; (b) PIR-GO in a frontal view, showing the aromatic rings; (c) PIR-GO in a side view, to better visualize the interaction of the methyl hydrogen with the GO sheet (distance of 2.30 Å). All of these interactions produced a very favored adsorption complex with an adsorption energy of 19.356 kcal mol^–1^.

As expected, the favored orientation of the PIR molecule over the pristine G-nanosheet was in parallel, by a π–π stacking interaction between the two conjugated systems, that is, the molecule and the G-nanosheet. As it is known this interaction is observed by the relative position of the benzene rings of the adsorbate molecule and the hexagons of the G-nanosheet; this is a noncovalent interaction associated with a physisorption process.^39–41^ In Figure 5a, it can be observed that the closer distances between an aromatic hydrogen of the PIR molecule and a carbon atom of the G-nanosheet are 3.00 Å in the pyridyl ring and 3.04 Å in the benzene ring; these distances are much longer than the bond distances C_arom_–H_arom_ of 1.1 Å, which should be related with a physisorption event.

As a result of parallel orientation of the PIR molecule, other interactions involving an aromatic hydrogen were found; though the intramolecular hydrogen bond of the PIR molecule (>C=O···H–O) was maintained at 1.65 Å of distance, that same hydrogen is forming a new noncovalent interaction with a carbon of the G-nanosheet (O–H···C_sheet_), at a distance of 2.92 Å.

These two interactions produced a very favored adsorption complex PIR-G, with an adsorption energy of 20.809 kcal mol^–1^ (∼87 kJ mol^–1^). According to this energy, the adsorption process could be near to a chemisorption event. Sometimes, differentiating between physisorption and chemisorption is not easy, especially since intermediate behaviors can occur.^42^ There is an important highlight that the π–π stacking interaction is responsible for the selective adsorption ability of graphene for the removal of organic molecules containing benzene rings in wastewater, which makes it an interesting and promising adsorbent material.^43^

On the other hand, Figure 5b,c presents the most favored geometry for the adsorption complex PIR-GO in frontal and side views, respectively. Note that in this complex, the PIR molecule has tilted, to favor its adsorption through the following four interactions which are listed: (i) A hydrogen bond involving the carbonyl group of the molecule and an –OH of the sheet, which belongs to a carboxyl group, with a distance of 1.96 Å; (ii) a second hydrogen bond involving the –OH group of the molecule and a carbonyl group, which belongs to the same carboxyl group, with a distance of 2.31 Å; (iii) three interactions mediated by aromatic hydrogens involving > C_arom_–H_arom_···O–H and > C_arom_–H_arom_···O_epox_ with adsorption distances of 2.21 Å and 2.30 Å, respectively. These noncovalent interactions mediated by aromatic hydrogens have been detected as very characteristic when the adsorption processes involve molecules with heteroatoms, such as the AINE-type drugs interacting with GO-nanosheets.

You can notice that GO-nanosheets are involved in five interactions with the PIR molecule; of these, only one is a hydrogen bond, as the rest correspond to noncovalent interactions where one aromatic hydrogen and three hydrogens from the methyl group interact either with an epoxy oxygen or with an oxygen from an OH group, both from the GO nanosheet. These five interactions are contact points molecule-GO-nanosheet, responsible of an adsorption energy of 19.356 kcal mol^–1^ (∼81 kJ mol^–1^), which is slightly less than that obtained with the G-nanosheet.

This result is indicating that the adsorption of the PIR molecule over the G-nanosheet is something stronger than the GO-nanosheet. This leads us to think that the π–π stacking interaction by generating more contact points between the molecule and the nanosheet, due to the orientation of the aromatic ring of the molecule, would leave the molecule more anchored to the nanosheet even though it would be further away from it (remember that the adsorption distance for this interaction is approximately 3 Å).

Figure 6 presents the mapping of the Frontier orbitals (HOMO and LUMO) and the MEP for the two adsorption complexes analyzed.

**Figure 6:**
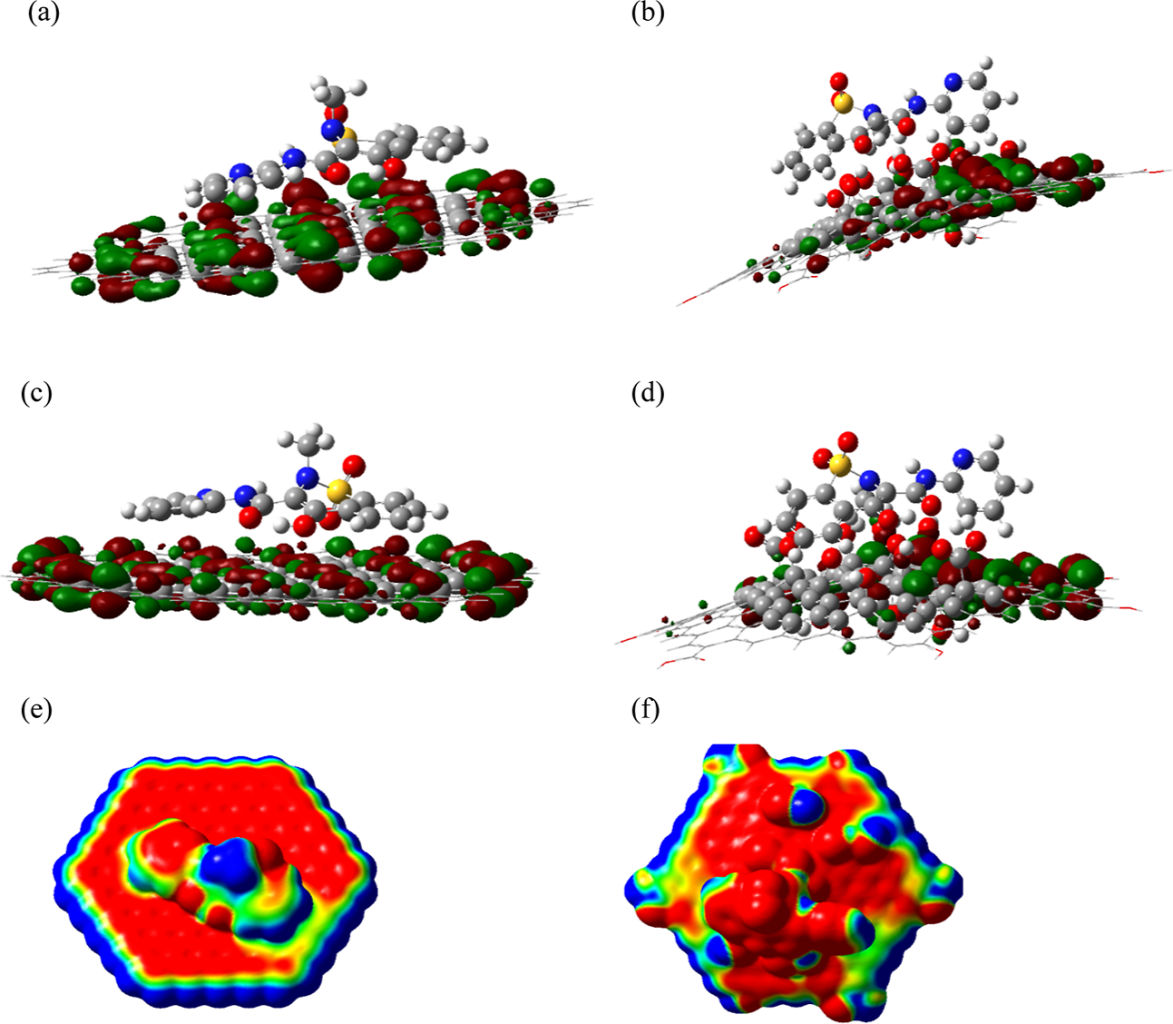
Maps of Frontier orbitals and the MEPs of the two studied adsorption complexes: (a) HOMO orbital of PIR-G; (b) HOMO orbital of PIR-GO; (c) LUMO orbital of PIR-G; (d) LUMO orbital of PIR-GO; (e) MEP of PIR-G; (f) MEP of PIR-GO. Note that on the left appear the images corresponding to the PIR-G complex, and on the right, those corresponding to the PIR-GO complex.

It can be observed that both complexes kept their Frontier orbitals only in the sheet, which can be related to a high tendency to move the electronic charge on the sheet and therefore, change its electronic properties, and the HOMO–LUMO gap, as the result of the adsorption process; these values were 706 and 327 meV for the G and GO sheets, respectively. Finally, the MEP maps show continuous electronic charge zones between the surfaces and the molecule, indicating donor–acceptor interactions in both directions and, therefore, an adsorption process.

Table 2 presents the thermochemistry of the adsorption processes for the PIR-G and PIR-GO complexes. To obtain the thermodynamic parameters, the difference between the adsorption complex (final state) and the sum of the free PIR molecule and free nanosheet (initial states) was considered.

**Table 2:** Thermodynamics Parameter for the Adsorption Process; Δ*H*_ads_ and Δ*H*_ads_ Are Expressed in kcal mol-1 and Δ*S*_ads_ in cal mol-1 < K-1

parameters	PIR-G	PIR-GO
Δ*H*_ads_	–19.33	–17.89
Δ*G*_ads_	–1.92	–1.52
Δ*S*_ads_	–58.43	–54.94

You can notice that in both systems, the Δ*G*_ads_ and Δ*H*_ads_ are negative, which indicates spontaneous and exothermic processes; besides, in total agreement with the adsorption energies discussed above, the PIR-G adsorption complex shows slightly more negative values. With respect to the entropy, the negative values mean that in the adsorption process the disorder decreases because the molecule is now attached to the nanosheet. The more negative values were observed in the PIR-G complex; this result may be related to the parallel configuration adopted by this molecule, which generates more contact points with the nanosheet and consequently less configurational flexibility to fit over it.

### 3.4 Contribution of the Adsorption Process to the Degradation of PCT

To evaluate how much the GO can help to degrade the PIR molecule, Table 2 presents some relevant percentages of elongation of the bonds in the PIR molecule (higher than 0.1%) considering the bond distances before and after the adsorption process. From Table 3 some interesting comments can be made: first, the elongations associated with polar bonds such as carbon–nitrogen and carbon–oxygen bonds are greater than those with carbon–carbon bonds. The more polarized bonds are easier to weaken in an adsorption process; and second, GO-sheet weakened the PIR molecule’s bonds more than graphene; this could be a good indicator of GO’s better properties as an adsorbent material.

**Table 3:** Percentages of Elongation of the Bonds in the PIR Molecule during the Adsorption Process (the Numbers of the Atoms and Bonds Are Related Figure 4)­^a^

^a^bonds in PIR	C1–C2	C13–C14	O22–C2	O21–C9	N10–C11	N16–C15	N20–C23
% elongation	0.13	0.13	0.42	0.14	0.58	0.26	0.21

a For the identification of bonds in PIR by their numbering, see Figure 2.

As an interesting aspect to highlight, these elongations can be related to some fragments or degradation products of this molecule, identified by mass spectrometric techniques;^44^ for example, from Table 2 we can say that.

(i) The breaking of the N10–C11 bond is related to the release of the pyridine ring cation of *m*/*e* 78, followed by the breaking of this ring, starting with the N16–C15 and C13–C14 bonds.
(ii) The breaking of the C1–C2 bond, which simultaneously with S17–N20 could produce a protonated fragment of *m*/*e* 121.04.
(iii) The breaking of O22–C2 could imply the release of a water molecule due to a previous protonation of the –OH group.
(iv) The breaking of the N20–C23 bond could be related to the release of the methyl group.

From a theoretical point of view, these last results are relevant to evaluating the contribution of the adsorbent surfaces in emergent contaminant degradation since the greater the elongation, the greater the probability of breaking bonds in the molecule, which could even trigger other breaks that would further favor the degradation of this drug.

## 4 Conclusions

A study of the adsorption complexes PIR-G and PIR-GO was made, considering descriptors like the adsorption energies and interaction distances between the molecule and sheet; also, some relevant elongations of the bonds in the PIR molecule as the result of the adsorption process were analyzed. The results allowed us to conclude that the adsorption process of the PIR molecule on G and GO sheets is a highly favored chemisorption but much more favored on the GO sheet. In this way, it can be confirmed that the heterogeneity of the GO sheet, due to the functional groups present and regulated by structural parameters like the C/O ratio and the oxygen percentage, has a great influence on its adsorption capacity and stability.

For the graphene sheet, the main interaction involved in this chemisorption process was a π-stacking interaction between both conjugate systems (molecule-sheet). For the graphene oxide sheet, hydrogen bonds involving the > C–O, –OH, and > NH groups of the molecule with the –OH and epoxide groups of graphene oxide were identified. In both sheets, noncovalent interactions mediated by aromatic hydrogens and methyl hydrogens, with an arrangement very similar to a hydrogen bond, were found.

The present theoretical study complements experimental studies that have confirmed graphene oxide as an effective adsorbent material of the piroxicam molecule and perhaps of other NSAID-type drugs, since it can help their degradation; in this way, we think that this nanomaterial could be included as one more element of current hybrid methodologies designed to improve water quality.

## Supplementary Material



## Notes

Coordinates of the initial and optimized structures for the GO-sheet and an adsorption complex (PDF)
