# Supplementary material for: Piroxicam Adsorption over Graphene OXIDE Nanosheets: A DFT-ONIOM Study
Source: ACS Omega. 2026 May 6;11(19):28246–54. doi: 10.1021/acsomega.5c13355 (PMC13191552; doi:10.1021/acsomega.5c13355)
Supplement: Supplementary file 1 [file ao5c13355_si_001.pdf]

Supporting Information

Title: "PIROXICAM ADSORPTION OVER GRAPHENE OXIDE NANOSHEETS: A DFT-ONIOM STUDY"

*[a]Alejandro M. Velázquez-García, [b]Sandy M. Pacheco-Ortin, [b]Benjamín Velasco-Bejarano, [b]Roberto Mejía-Olvera, [a,b]Esther AgacinoValdés**

[a]Centro de Investigaciones Teóricas, Facultad de Estudios Superiores Cuautitlán, Universidad Nacional Autónoma de México, Cuautitlán Izcalli, CP 54740, Edo. México, México.

[b]Departamento de Química, Facultad de Estudios Superiores Cuautitlán, Universidad Nacional Autónoma de México, Cuautitlán Izcalli, CP 54740, Estado de México, México. México

CONTENT

(A) CARTESIAN COORDINATES FOR THE INITIAL (INPUT) AND OPTIMIZED (OUTPUT) GEOMETRIES OF THE GRAPHENE OXIDE SHEET USING ONIOM METHODOLOGY

A-1 Cartesian coordinates of the initial geometry of the Graphene Oxide sheet (INPUT FILE).

A-2 Cartesian coordinates of the optimized geometry of the Graphene Oxide sheet (OUTPUT FILE)

(B) CARTESIAN COORDINATES OF THE INITIAL (INPUT) AND OPTIMIZED (OUTPUT) GEOMETRIES OF THE PIROXICAM-GRAPHENE OXIDE ADSORPTION COMPLEX USING ONIOM METHODOLOGY

B-1 Cartesian coordinates of the initial geometry of the Piroxicam-Graphene Oxide adsorption complex (INPUT FILE)

B-2 Cartesian coordinates of the optimized geometry of the Piroxicam-Graphene Oxide adsorption complex (OUTPUT FILE)

(A) CARTESIAN COORDINATES FOR THE INITIAL (INPUT) AND OPTIMIZED (OUTPUT) GEOMETRIES OF THE GRAPHENE OXIDE SHEET USING ONIOM METHODOLOGY

A-1 Cartesian coordinates of the initial geometry of the Graphene Oxide sheet (INPUT FILE)

Symbolic Z-matrix:

Charge = 0 Multiplicity = 1 for low level calculation on real system.
Charge = 0 Multiplicity = 1 for high level calculation on model system.
Charge = 0 Multiplicity = 1 for low level calculation on model system.

C	0	-1.1201	-3.70755	0.97797	H
C	0	0.11595	-2.86044	1.05138	H
C	0	-0.03541	-1.38407	0.98302	H
C	0	-1.2336	-0.79684	1.04832	H
C	0	-2.46623	-1.54483	0.59162	H
C	0	-2.41285	-3.04161	0.76506	H
C	0	1.1338	-0.6102	0.50622	H
C	0	-1.40588	0.66345	1.40377	H
C	0	-0.14361	1.49121	1.1343	H
C	0	1.13571	0.83799	0.79166	H
C	0	-0.26961	2.93706	0.85714	H
C	0	-1.50285	3.50205	0.82082	H
C	0	-2.71457	2.69627	0.75893	H
C	0	-2.67584	1.29864	0.89028	H
C	0	-3.82893	0.52778	0.80483	H
C	0	-3.76701	-0.9561	1.14815	H
C	0	-4.94015	-1.74119	0.57673	H
C	0	-5.06819	1.14432	0.56534	H
C	0	-0.96967	-5.10095	0.53542	H
C	0	-3.5236	-3.77398	0.52918	H
C	0	-1.64224	4.99242	1.0073	H
C	0	-3.95305	3.32275	0.52448	H
C	0	1.39325	-3.48822	0.68203	H
C	0	2.60774	-2.60865	0.75078	H
C	0	2.38323	-1.25817	0.10393	H
C	0	1.48989	-4.83342	0.52666	H
C	0	3.87627	-3.26809	0.30188	H
C	0	3.61196	-0.43726	-0.27106	H
C	0	3.49365	1.02378	0.1053	H
C	0	2.30522	1.63707	0.363	H
C	0	4.92034	-1.0612	0.16048	H
C	0	4.70066	1.79687	-0.00069	H
C	0	0.94201	3.69306	0.6166	H
C	0	0.81776	5.13857	0.4894	H
C	0	2.17474	3.07719	0.4137	H

C	0	3.36309	3.85365	0.15908	H				
C	0	-5.11491	2.54295	0.46755	H				
C	0	-4.81988	-3.0993	0.38405	H				
C	0	-6.17988	-1.08037	0.32819	H				
C	0	-6.27208	0.32906	0.35395	H				
C	0	-0.39489	5.74524	0.61595	H				
C	0	-2.89273	5.54422	0.34504	H				
C	0	2.02806	5.90868	0.21669	H				
C	0	4.60627	3.21695	-0.00537	H				
C	0	3.28088	5.26738	0.07824	H				
C	0	5.94376	1.15636	-0.06306	H				
C	0	6.05433	-0.28015	0.08358	H				
C	0	4.9891	-2.48574	0.23805	H				
C	0	3.938	-4.70837	0.27421	H				
C	0	2.80393	-5.4572	0.36651	H				
C	0	0.28422	-5.66012	0.43037	H				
C	0	-3.42905	-5.20827	0.28482	H				
C	0	-2.15941	-5.84232	0.2562	H				
C	0	5.79684	3.99455	-0.20763	L	H	44	0.	0.
C	0	4.46017	6.03815	-0.1863	L	H	45	0.	0.
C	0	1.95573	7.29	0.03148	H				
C	0	-0.51821	7.24279	0.64855	H				
C	0	-2.92359	6.94414	-0.04245	H				
C	0	-5.27585	5.33878	-0.16958	H				
C	0	-6.46301	3.23072	0.4682	H				
C	0	-7.49076	0.9437	0.0084	H				
C	0	-7.32358	-1.85807	-0.04113	L	H	39	0.	0.
C	0	-5.97476	-3.90289	0.02635	L	H	38	0.	0.
C	0	-4.57328	-5.94843	-0.0509	L	H	52	0.	0.
C	0	-2.06298	-7.19699	-0.09184	H				
C	0	0.40443	-7.05387	0.07457	H				
C	0	2.90592	-6.96086	0.47185	H				
C	0	5.2454	-5.32131	0.05447	H				
C	0	6.32761	-3.12401	0.50389	H				
C	0	7.37115	-0.90569	-0.03375	H				
C	0	7.14286	1.94036	-0.25195	L	H	46	0.	0.
C	0	4.37127	7.43177	-0.35202	L				
C	0	3.12003	8.04763	-0.27493	L	H	56	0.	0.
C	0	0.68252	7.95161	0.08045	H				
C	0	-1.79759	7.73214	0.00896	H				
C	0	-4.18224	7.50998	-0.47788	H				
C	0	-5.31761	6.73685	-0.53334	H				
C	0	-6.42656	4.60392	-0.15091	H				
C	0	-7.5809	2.37533	-0.06284	H				
C	0	-8.63468	0.1703	-0.33832	L	H	61	0.	0.
C	0	-8.53739	-1.23101	-0.33559	L				
C	0	-7.21278	-3.27216	-0.14997	L				
C	0	-5.85674	-5.29094	-0.15012	L				

C	0	-3.21794	-7.95451	-0.37252	L	H	65	0.	0.
C	0	-4.46601	-7.31852	-0.3458	L				
C	0	-0.74959	-7.80472	-0.17992	H				
C	0	1.67078	-7.67272	-0.03565	H				
C	0	8.51127	-0.11953	-0.33248	L	H	70	0.	0.
C	0	8.38304	1.31531	-0.397	L				
C	0	7.04508	3.34801	-0.33861	L				
C	0	5.70837	5.38593	-0.3053	L				
C	0	7.48771	-2.30001	0.02187	H				
C	0	6.37576	-4.5698	0.04733	H				
C	0	5.29462	-6.74302	-0.23189	H				
C	0	4.16999	-7.49849	-0.16548	H				
C	0	-3.08405	-9.36228	-0.71235	L				
C	0	-5.65775	-8.06949	-0.65542	L				
C	0	-6.99821	-6.05062	-0.48027	L				
C	0	-8.35544	-4.04869	-0.49092	L				
C	0	-9.68506	-2.0321	-0.6752	L				
C	0	-9.85354	0.82461	-0.72356	L				
C	0	-8.73727	2.96409	-0.51114	L	H	79	0.	0.
C	0	-7.69407	5.18123	-0.58393	L	H	78	0.	0.
C	0	-6.60461	7.32154	-0.91667	L	H	77	0.	0.
C	0	-4.24618	8.93767	-0.80649	L	H	76	0.	0.
C	0	-1.85821	9.14408	-0.36639	L	H	75	0.	0.
C	0	0.57623	9.28388	-0.26075	L	H	74	0.	0.
C	0	2.98319	9.44691	-0.52306	L				
C	0	5.57293	8.21018	-0.62753	L				
C	0	6.8854	6.15958	-0.55165	L				
C	0	8.21225	4.12708	-0.5668	L				
C	0	9.53444	2.1013	-0.64078	L				
C	0	9.74997	-0.74666	-0.57417	L				
C	0	8.70197	-2.91563	-0.30929	L	H	92	0.	0.
C	0	7.67884	-5.15377	-0.29329	L	H	93	0.	0.
C	0	6.60522	-7.32856	-0.55224	L	H	94	0.	0.
C	0	4.21569	-8.92543	-0.50929	L	H	95	0.	0.
C	0	1.7627	-9.01785	-0.42017	L	H	87	0.	0.
C	0	-0.65288	-9.1687	-0.54453	L	H	86	0.	0.
C	0	-6.8605	-7.45954	-0.7006	L				
C	0	-8.22803	-5.41808	-0.6299	L				
C	0	-9.59266	-3.38088	-0.72462	L				
C	0	-10.92173	-1.3544	-0.98024	L				
C	0	-11.01769	-0.02241	-1.01805	L				
C	0	-9.87216	2.18453	-0.82059	L				
C	0	-8.78555	4.39947	-0.69907	L				
C	0	-7.71677	6.57788	-0.93858	L				
C	0	-6.62861	8.73622	-1.26204	L				
C	0	-5.53232	9.48338	-1.20791	L				
C	0	-3.13998	9.69247	-0.7366	L				
C	0	-0.72743	9.88813	-0.40311	L				

C	0	1.75292	10.02724	-0.53908	L
C	0	4.18148	10.21219	-0.78347	L
C	0	5.39662	9.65696	-0.82337	L
C	0	6.7662	7.5808	-0.70067	L
C	0	8.09604	5.52906	-0.66723	L
C	0	9.43977	3.47399	-0.71197	L
C	0	10.79286	1.4386	-0.83848	L
C	0	10.91302	0.1027	-0.82588	L
C	0	9.8113	-2.14116	-0.58543	L
C	0	8.7618	-4.36868	-0.41192	L
C	0	7.71288	-6.57991	-0.56414	L
C	0	6.63095	-8.76154	-0.82346	L
C	0	5.52281	-9.49676	-0.80437	L
C	0	3.07785	-9.63119	-0.58891	L
C	0	0.60443	-9.74395	-0.66673	L
C	0	-1.85148	-9.9169	-0.78912	L
C	0	-4.33315	-10.1006	-0.96692	L
C	0	-5.51689	-9.48278	-0.93317	L
H	0	-6.40613	-10.04335	-1.12648	L
H	0	-7.74219	-8.02495	-0.93315	L
H	0	-9.08953	-6.00317	-0.88802	L
H	0	-10.45396	-3.9703	-0.97317	L
H	0	-11.7817	-1.95102	-1.19605	L
H	0	-10.76558	2.67665	-1.13001	L
H	0	-9.71664	4.82796	-1.01622	L
H	0	-8.64612	7.02055	-1.22998	L
H	0	-5.56857	10.52542	-1.46104	L
H	0	-3.18187	10.73543	-0.98461	L
H	0	-0.77811	10.92716	-0.66393	L
H	0	1.65944	11.07143	-0.76766	L
H	0	4.07973	11.2637	-0.94566	L
H	0	7.65158	8.14364	-0.89032	L
H	0	8.97933	6.1094	-0.85128	L
H	0	10.32255	4.05555	-0.89503	L
H	0	11.65786	2.04054	-1.01556	L
H	0	10.74024	-2.61256	-0.81045	L
H	0	9.70897	-4.80202	-0.66943	L
H	0	8.65573	-7.0334	-0.78914	L
H	0	5.56408	-10.5465	-1.02388	L
H	0	3.10553	-10.67133	-0.85032	L
H	0	0.68157	-10.77324	-0.95946	L
H	0	-1.75494	-10.94799	-1.04281	L
C	0	-12.32813	0.56913	-1.36782	L
O	0	-12.60049	1.7406	-1.4846	L
O	0	-13.28206	-0.37652	-1.56976	L
H	0	-14.13254	0.02574	-1.79677	L
C	0	-4.31711	-11.5481	-1.27507	L
O	0	-3.35332	-12.27068	-1.36729	L

O	0	-5.5659	-12.04956	-1.46452	L
H	0	-5.53655	-12.99603	-1.66439	L
C	0	6.55494	10.53824	-1.08696	L
O	0	7.7185	10.22113	-1.1655	L
O	0	6.18971	11.83685	-1.2473	L
H	0	6.95887	12.39892	-1.41784	L
C	0	12.25459	-0.47258	-1.07005	L
O	0	12.55905	-1.64082	-1.12155	L
O	0	13.19916	0.4865	-1.25093	L
H	0	14.06989	0.09599	-1.41229	L
O	0	-7.86981	9.188	-1.627	L
H	0	-7.8874	10.12596	-1.85147	L
O	0	7.88421	-9.24226	-1.09923	L
H	0	7.90182	-10.1887	-1.28499	L
O	0	0.81897	1.16293	2.14663	H
O	0	2.78957	-2.23528	2.12552	H
H	0	3.44218	-2.83355	2.52441	H
O	0	-1.62923	0.68449	2.82879	H
H	0	-0.78776	0.42036	3.23475	H
C	0	-4.00627	4.76675	0.25917	H
C	0	-6.76884	3.40139	2.00354	H
O	0	-7.48757	2.67396	2.62969	H
O	0	-6.06383	4.40348	2.53647	H
H	0	-6.2086	4.37727	3.49483	H
C	0	6.40704	-3.25148	2.06886	H
O	0	5.4449	-3.37613	2.78395	H
O	0	7.65799	-3.27754	2.52181	H
H	0	7.61029	-3.43379	3.47852	H
O	0	-0.57707	7.57466	2.06719	H
H	0	-0.73755	8.52644	2.13314	H
O	0	2.98154	-7.19925	1.88713	H
H	0	2.94129	-8.15667	2.01785	H
O	0	-0.49421	-3.4035	2.22333	H
O	0	-3.84808	-1.13486	2.54698	H
H	0	-3.16604	-0.57009	2.95266	H
O	0	-1.84341	5.11965	2.42756	H
H	0	-1.58375	6.02534	2.66484	H
O	0	1.35752	-1.04994	-0.83862	H
O	0	3.62786	-0.50674	-1.70475	H
H	0	4.44052	-0.0766	-2.00247	H
O	0	-2.40816	-1.28143	-0.81976	H
H	0	-3.20792	-1.65191	-1.22176	H

A-2 Cartesian coordinates of the optimized geometry of the Graphene Oxide sheet (OUTPUT FILE)

Center Number	Atomic Number	Atomic Type	Coordinates (Angstroms) X	Y	Z
1	6	0	1.056442	3.718446	0.977467
2	6	0	-0.163374	2.855798	1.060390
3	6	0	0.011507	1.379259	1.003225
4	6	0	1.219424	0.808752	1.058724
5	6	0	2.436213	1.578095	0.588323
6	6	0	2.361127	3.073849	0.768616
7	6	0	-1.149216	0.585048	0.534748
8	6	0	1.418532	-0.651990	1.408446
9	6	0	0.167992	-1.498856	1.144047
10	6	0	-1.124761	-0.867225	0.806161
11	6	0	0.313559	-2.946573	0.868830
12	6	0	1.565299	-3.491798	0.825464
13	6	0	2.757208	-2.663818	0.756673
14	6	0	2.696349	-1.263406	0.882609
15	6	0	3.837132	-0.474363	0.802322
16	6	0	3.748874	1.008873	1.138061
17	6	0	4.910471	1.815202	0.577097
18	6	0	5.090573	-1.067601	0.569140
19	6	0	0.884761	5.110773	0.536718
20	6	0	3.460682	3.823754	0.534975
21	6	0	1.728028	-4.976234	1.017041
22	6	0	4.012606	-3.265621	0.531758
23	6	0	-1.454015	3.461208	0.692443
24	6	0	-2.656166	2.568166	0.775497
25	6	0	-2.413314	1.214995	0.142338
26	6	0	-1.571495	4.803389	0.525065
27	6	0	-3.935580	3.202633	0.316362
28	6	0	-3.627774	0.376307	-0.246488
29	6	0	-3.489740	-1.083576	0.126672
30	6	0	-2.283810	-1.679708	0.376656
31	6	0	-4.950108	0.983381	0.171499
32	6	0	-4.674814	-1.870161	0.031535
33	6	0	-0.878301	-3.716009	0.648983
34	6	0	-0.729476	-5.145572	0.506713
35	6	0	-2.138114	-3.110033	0.439372
36	6	0	-3.296526	-3.895529	0.195390
37	6	0	5.161192	-2.465623	0.479530

38	6	0	4.768362	3.171489	0.388012
39	6	0	6.161549	1.174797	0.330482
40	6	0	6.278571	-0.232081	0.359464
41	6	0	0.503259	-5.741891	0.603795
42	6	0	2.992579	-5.506026	0.365550
43	6	0	-1.928470	-5.925189	0.232959
44	6	0	-4.562815	-3.274793	0.034187
45	6	0	-3.185971	-5.309681	0.107744
46	6	0	-5.945728	-1.244921	-0.037621
47	6	0	-6.074712	0.185746	0.099363
48	6	0	-5.039352	2.404252	0.250990
49	6	0	-4.018446	4.641241	0.269665
50	6	0	-2.894767	5.407684	0.357720
51	6	0	-0.377842	5.649697	0.429267
52	6	0	3.341991	5.257149	0.290669
53	6	0	2.063580	5.869513	0.258712
54	6	0	-5.735604	-4.069438	-0.160067
55	6	0	-4.361060	-6.100623	-0.161742
56	6	0	-1.828737	-7.315931	0.020309
57	6	0	0.641174	-7.233350	0.635770
58	6	0	3.045346	-6.905056	-0.035653
59	6	0	5.372745	-5.257886	-0.162636
60	6	0	6.522294	-3.131173	0.487313
61	6	0	7.509187	-0.826340	0.016874
62	6	0	7.291501	1.973267	-0.035250
63	6	0	5.908806	3.994242	0.032852
64	6	0	4.474892	6.015108	-0.046790
65	6	0	1.944697	7.221652	-0.095602
66	6	0	-0.521683	7.041055	0.072448
67	6	0	-3.021121	6.909554	0.463233
68	6	0	-5.334718	5.233384	0.039027
69	6	0	-6.387511	3.026265	0.499393
70	6	0	-7.395277	0.793490	-0.025257
71	6	0	-7.109807	-2.042607	-0.215252
72	6	0	-4.252877	-7.488003	-0.353305
73	6	0	-2.977739	-8.087558	-0.298762
74	6	0	-0.550954	-7.950790	0.051473
75	6	0	1.932693	-7.707372	0.003964
76	6	0	4.312929	-7.444736	-0.485816
77	6	0	5.434952	-6.654104	-0.538311
78	6	0	6.509280	-4.502969	-0.140722
79	6	0	7.623292	-2.255996	-0.050969
80	6	0	8.641248	-0.031513	-0.326646
81	6	0	8.517850	1.368370	-0.326362

82	6	0	7.156898	3.384718	-0.144965
83	6	0	5.769008	5.379549	-0.145211
84	6	0	3.087913	7.998377	-0.379519
85	6	0	4.346796	7.382154	-0.348115
86	6	0	0.622320	7.806810	-0.187829
87	6	0	-1.797576	7.639744	-0.043426
88	6	0	-8.526846	-0.013557	-0.316688
89	6	0	-8.373158	-1.437083	-0.368514
90	6	0	-6.985584	-3.449441	-0.293239
91	6	0	-5.619339	-5.469363	-0.265853
92	6	0	-7.530047	2.182299	0.019058
93	6	0	-6.453524	4.464299	0.030746
94	6	0	-5.404806	6.652707	-0.252636
95	6	0	-4.290705	7.425947	-0.182359
96	6	0	2.933576	9.404065	-0.728717
97	6	0	5.525277	8.150871	-0.662780
98	6	0	6.896623	6.157047	-0.479100
99	6	0	8.286395	4.179610	-0.487245
100	6	0	9.651309	2.188684	-0.665156
101	6	0	9.875150	-0.664002	-0.707547
102	6	0	8.790103	-2.824788	-0.499264
103	6	0	7.787196	-5.055044	-0.581115
104	6	0	6.732869	-7.214625	-0.930048
105	6	0	4.397380	-8.868477	-0.835240
106	6	0	2.009989	-9.110859	-0.410345
107	6	0	-0.426426	-9.280740	-0.327792
108	6	0	-2.824200	-9.469339	-0.581827
109	6	0	-5.432202	-8.283350	-0.633425
110	6	0	-6.775105	-6.257901	-0.514985
111	6	0	-8.140705	-4.248644	-0.521292
112	6	0	-9.507316	-2.243445	-0.607872
113	6	0	-9.780758	0.589711	-0.562170
114	6	0	-8.756233	2.775818	-0.319493
115	6	0	-7.763072	5.026283	-0.316993
116	6	0	-6.723873	7.218405	-0.583877
117	6	0	-4.356117	8.847983	-0.543992
118	6	0	-1.909086	8.978456	-0.447134
119	6	0	0.503932	9.165728	-0.564291
120	6	0	6.739713	7.562474	-0.706538
121	6	0	8.139077	5.547077	-0.630523
122	6	0	9.537218	3.536762	-0.719439
123	6	0	10.901399	1.536476	-0.962694
124	6	0	11.027571	0.205111	-0.994535
125	6	0	9.911559	-2.024636	-0.807706

126	6	0	8.868672	-4.259686	-0.693004
127	6	0	7.831611	-6.449930	-0.946165
128	6	0	6.775734	-8.627819	-1.290642
129	6	0	5.690331	-9.392045	-1.243105
130	6	0	3.300543	-9.639546	-0.783786
131	6	0	0.888810	-9.861316	-0.488978
132	6	0	-1.578710	-10.035674	-0.621253
133	6	0	-4.006262	-10.251513	-0.853164
134	6	0	-5.234517	-9.720003	-0.866318
135	6	0	-6.641956	-7.664597	-0.684627
136	6	0	-8.008729	-5.639999	-0.625543
137	6	0	-9.382774	-3.619657	-0.671498
138	6	0	-10.778501	-1.615688	-0.806533
139	6	0	-10.929085	-0.279665	-0.801284
140	6	0	-9.856611	1.985141	-0.587191
141	6	0	-8.837704	4.226891	-0.435237
142	6	0	-7.819172	6.449800	-0.595574
143	6	0	-6.767518	8.651559	-0.864063
144	6	0	-5.668082	9.399885	-0.849652
145	6	0	-3.229606	9.571228	-0.632082
146	6	0	-0.761697	9.721160	-0.698615
147	6	0	1.689350	9.932734	-0.811818
148	6	0	4.172332	10.161272	-0.990257
149	6	0	5.364842	9.557714	-0.952297
150	1	0	6.253900	10.116668	-1.149789
151	1	0	7.611959	8.139466	-0.943803
152	1	0	8.989830	6.145700	-0.890786
153	1	0	10.387171	4.141537	-0.967868
154	1	0	11.741413	2.161772	-1.174637
155	1	0	10.805670	-2.511219	-1.119464
156	1	0	9.805981	-4.670726	-1.013020
157	1	0	8.774989	-6.859690	-1.240146
158	1	0	5.746406	-10.429362	-1.508951
159	1	0	3.356363	-10.677371	-1.049389
160	1	0	0.951030	-10.892014	-0.778095
161	1	0	-1.472226	-11.071431	-0.877579
162	1	0	-3.870485	-11.293922	-1.043932
163	1	0	-7.524006	-8.230230	-0.877091
164	1	0	-8.882697	-6.233602	-0.810536
165	1	0	-10.253547	-4.219354	-0.851991
166	1	0	-11.621760	-2.249605	-0.974664
167	1	0	-10.785243	2.451595	-0.817525
168	1	0	-9.789106	4.646513	-0.697855
169	1	0	-8.775294	6.871972	-0.824651

170	1	0	-5.726061	10.446497	-1.076050
171	1	0	-3.271653	10.607126	-0.906175
172	1	0	-0.855657	10.745694	-1.001460
173	1	0	1.563591	10.957596	-1.072947
174	6	0	12.356039	-0.355809	-1.326037
175	8	0	12.645231	-1.531779	-1.427873
176	8	0	13.289642	0.600325	-1.518021
177	1	0	14.160980	0.230443	-1.741354
178	6	0	4.139651	11.606405	-1.306029
179	8	0	3.154250	12.315414	-1.363243
180	8	0	5.367307	12.116602	-1.541788
181	1	0	5.342858	13.066912	-1.747061
182	6	0	-6.376396	-10.621323	-1.143935
183	8	0	-7.550498	-10.311746	-1.187046
184	8	0	-5.990281	-11.895515	-1.358762
185	1	0	-6.738022	-12.487249	-1.548688
186	6	0	-12.285862	0.263894	-1.035189
187	8	0	-12.603019	1.435752	-1.079079
188	8	0	-13.206617	-0.706582	-1.207735
189	1	0	-14.098441	-0.352142	-1.366205
190	8	0	8.022301	-9.066923	-1.667049
191	1	0	8.037563	-10.005218	-1.911285
192	8	0	-8.027015	9.123021	-1.146048
193	1	0	-8.047739	10.077329	-1.317621
194	8	0	-0.796704	-1.183534	2.162311
195	8	0	-2.840036	2.211185	2.158336
196	1	0	-3.452333	2.851623	2.560322
197	8	0	1.654790	-0.676240	2.832970
198	1	0	0.846838	-0.344392	3.259923
199	6	0	4.092616	-4.709145	0.272987
200	6	0	6.825239	-3.299108	2.019543
201	8	0	7.537000	-2.548579	2.643115
202	8	0	6.151181	-4.307684	2.560119
203	1	0	6.311012	-4.309861	3.520869
204	6	0	-6.478094	3.178624	2.067980
205	8	0	-5.514080	3.416191	2.758104
206	8	0	-7.716077	3.086673	2.521423
207	1	0	-7.712637	3.253366	3.482039
208	8	0	0.691894	-7.592254	2.055317
209	1	0	0.952977	-8.523720	2.125851
210	8	0	-3.112729	7.138032	1.885489
211	1	0	-3.100923	8.096554	2.033109
212	8	0	0.439992	3.413845	2.238031
213	8	0	3.811808	1.192135	2.547359

214	1	0	3.127781	0.621542	2.946897
215	8	0	1.934605	-5.119486	2.455140
216	1	0	1.748318	-6.054476	2.653025
217	8	0	-1.384238	1.031151	-0.810754
218	8	0	-3.632740	0.455477	-1.682130
219	1	0	-4.438840	0.025931	-2.006620
220	8	0	2.367129	1.316978	-0.824044
221	1	0	3.178914	1.653140	-1.238880

(B) CARTESIAN COORDINATES OF THE INITIAL (INPUT) AND OPTIMIZED (OUTPUT) GEOMETRIES OF THE PIROXICAM-GRAPHENE OXIDE ADSORPTION COMPLEX USING ONIOM METHODOLOGY

B-1 Cartesian coordinates of the initial geometry of the Piroxicam-Graphene Oxide adsorption complex (INPUT FILE)

```
Symbolic Z-matrix:
 Charge =  0 Multiplicity = 1 for low   level calculation on real  system.
 Charge =  0 Multiplicity = 1 for high  level calculation on model system.
 Charge =  0 Multiplicity = 1 for low   level calculation on model system.
 C      0    1.68624   3.30323   0.23584  H
 C      0    0.37664   2.5761    0.27708  H
 C      0    0.39369   1.08831   0.2899   H
 C      0    1.52872   0.39978   0.44308  H
 C      0    2.84346   1.01393   0.01374  H
 C      0    2.92226   2.51488   0.12951  H
 C      0   -0.82216   0.39738  -0.20167  H
 C      0    1.55293  -1.05625   0.85892  H
 C      0    0.22923  -1.77591   0.57502  H
 C      0   -0.97062  -1.02724   0.1546   H
 C      0    0.22946  -3.23824   0.36797  H
 C      0    1.40677  -3.91078   0.39038  H
 C      0    2.68846  -3.2221    0.33643  H
 C      0    2.77559  -1.823     0.41538  H
 C      0    3.99765  -1.16502   0.35523  H
 C      0    4.05961   0.32986   0.64882  H
 C      0    5.32454   0.98222   0.10791  H
 C      0    5.18137  -1.90185   0.18411  H
 C      0    1.68717   4.68114  -0.277    H
 C      0    4.10696   3.13363  -0.0675   H
 C      0    1.40495  -5.39964   0.6389   H
 C      0    3.86962  -3.96828   0.16882  H
 C      0   -0.81744   3.29382  -0.19458  H
 C      0   -2.10908   2.53495  -0.14751  H
 C      0   -1.99026   1.13003  -0.6943   H
 C      0   -0.78315   4.63132  -0.43142  H
 C      0   -3.28563   3.27892  -0.69732  H
 C      0   -3.27827   0.40605  -1.07462  H
 C      0   -3.31428  -1.032    -0.60402  H
 C      0   -2.19717  -1.73711  -0.27319  H
 C      0   -4.5355    1.17524  -0.7342   H
 C      0   -4.58647  -1.69272  -0.70162  H
 C      0   -1.03978  -3.89174   0.12646  H
 C      0   -1.04649  -5.34703   0.06786  H
 C      0   -2.20466  -3.17741  -0.13993  H
 C      0   -3.45529  -3.85253  -0.38244  H
 C      0    5.09995  -3.3014    0.12937  H
```

C	0	5.34114	2.33899	-0.1253	H				
C	0	6.50605	0.20109	-0.06504	H				
C	0	6.46349	-1.20947	0.00029	H				
C	0	0.10363	-6.05339	0.24743	H				
C	0	2.61452	-6.08827	0.0317	H				
C	0	-2.31717	-6.0151	-0.19572	H				
C	0	-4.62869	-3.11299	-0.61281	H				
C	0	-3.50331	-5.26965	-0.38937	H				
C	0	-5.76042	-0.94365	-0.84017	H				
C	0	-5.73757	0.50322	-0.79075	H				
C	0	-4.46763	2.60269	-0.74909	H				
C	0	-3.21283	4.71111	-0.82459	H				
C	0	-2.02137	5.36099	-0.70971	H				
C	0	0.49774	5.34068	-0.49648	H				
C	0	4.15816	4.56105	-0.36325	H				
C	0	2.9544	5.30346	-0.49835	H				
C	0	-5.88371	-3.78726	-0.79764	L	H	44	0.	0.
C	0	-4.74386	-5.9413	-0.64088	L	H	45	0.	0.
C	0	-2.36741	-7.40368	-0.31991	H				
C	0	0.09104	-7.55353	0.34133	H				
C	0	2.52561	-7.49824	-0.30771	H				
C	0	5.0189	-6.11987	-0.4173	H				
C	0	6.37678	-4.11148	0.19818	H				
C	0	7.63116	-1.9453	-0.27933	H				
C	0	7.73297	0.85821	-0.3996	L	H	39	0.	0.
C	0	6.58195	3.02134	-0.44383	L	H	38	0.	0.
C	0	5.38274	5.18267	-0.65226	L	H	52	0.	0.
C	0	3.0028	6.64474	-0.90335	H				
C	0	0.52597	6.7201	-0.92142	H				
C	0	-1.99548	6.86996	-0.71036	H				
C	0	-4.44549	5.41946	-1.15956	H				
C	0	-5.74624	3.39361	-0.57599	H				
C	0	-6.98907	1.23955	-0.96121	H				
C	0	-7.02823	-1.62214	-0.99521	L	H	46	0.	0.
C	0	-4.78026	-7.34364	-0.74043	L				
C	0	-3.59128	-8.06571	-0.61513	L	H	56	0.	0.
C	0	-1.15949	-8.17401	-0.22268	H				
C	0	1.33162	-8.17893	-0.25542	H				
C	0	3.73649	-8.18977	-0.69314	H				
C	0	4.9399	-7.52628	-0.73967	H				
C	0	6.23202	-5.49398	-0.3836	H				
C	0	7.58722	-3.38037	-0.3139	H				
C	0	8.85584	-1.29193	-0.59566	L	H	61	0.	0.
C	0	8.89279	0.11217	-0.62606	L				
C	0	7.76181	2.27311	-0.54419	L				
C	0	6.60369	4.40829	-0.66012	L				
C	0	4.23762	7.28672	-1.12994	L	H	65	0.	0.
C	0	5.41864	6.5452	-0.99768	L				

C	0	1.75775	7.35756	-1.11648	H					
C	0	-0.66956	7.43938	-1.15945	H					
C	0	-8.19496	0.54908	-1.21573	L	H	70	0.	0.	
C	0	-8.20311	-0.89386	-1.19095	L					
C	0	-7.06363	-3.03624	-0.99057	L					
C	0	-5.92438	-5.18423	-0.81698	L					
C	0	-6.97195	2.6421	-0.99645	H					
C	0	-5.64058	4.77912	-1.15096	H					
C	0	-4.34782	6.81318	-1.54313	H					
C	0	-3.1612	7.46751	-1.46648	H					
C	0	4.25285	8.68465	-1.53164	L					
C	0	6.69053	7.17527	-1.25544	L					
C	0	7.82634	5.04931	-0.94795	L					
C	0	8.98732	2.92986	-0.8462	L					
C	0	10.12502	0.7926	-0.93046	L					
C	0	10.02043	-2.06792	-0.91722	L					
C	0	8.69768	-4.08701	-0.7045	L	H	79	0.	0.	
C	0	7.45208	-6.19858	-0.76008	L	H	78	0.	0.	
C	0	6.17648	-8.23783	-1.07004	L	H	77	0.	0.	
C	0	3.67623	-9.62702	-0.97692	L	H	76	0.	0.	
C	0	1.27087	-9.60286	-0.58172	L	H	75	0.	0.	
C	0	-1.16801	-9.52246	-0.51274	L	H	74	0.	0.	
C	0	-3.57764	-9.48099	-0.80408	L					
C	0	-6.04424	-8.02163	-1.00115	L					
C	0	-7.16453	-5.85826	-1.04428	L					
C	0	-8.29643	-3.71536	-1.18987	L					
C	0	-9.42257	-1.58233	-1.39587	L					
C	0	-9.37178	1.27459	-1.50118	L					
C	0	-8.11989	3.34699	-1.37525	L	H	92	0.	0.	
C	0	-6.8722	5.46253	-1.55528	L	H	93	0.	0.	
C	0	-5.58188	7.49225	-1.96333	L	H	94	0.	0.	
C	0	-3.05524	8.86643	-1.89679	L	H	95	0.	0.	
C	0	-0.61534	8.76251	-1.61958	L	H	87	0.	0.	
C	0	1.80774	8.7048	-1.54868	L	H	86	0.	0.	
C	0	7.83231	6.45784	-1.21153	L					
C	0	8.99695	4.3014	-1.02001	L					
C	0	10.16434	2.14265	-1.00927	L					
C	0	11.30237	-0.00668	-1.16966	L					
C	0	11.27103	-1.34219	-1.17949	L					
C	0	9.9123	-3.42565	-0.98611	L					
C	0	8.61556	-5.52528	-0.85749	L					
C	0	7.35346	-7.60092	-1.07845	L					
C	0	6.07731	-9.65817	-1.37713	L					
C	0	4.91544	-10.29956	-1.33117	L					
C	0	2.50435	-10.27582	-0.90868	L					
C	0	0.07837	-10.24445	-0.61256	L					
C	0	-2.40398	-10.16845	-0.77983	L					
C	0	-4.83762	-10.14589	-1.04982	L					

C	0	-5.99709	-9.48589	-1.13068	L
C	0	-7.17421	-7.2905	-1.12217	L
C	0	-8.31035	-5.1256	-1.21151	L
C	0	-9.45679	-2.95915	-1.38663	L
C	0	-10.61451	-0.81693	-1.63912	L
C	0	-10.60942	0.52202	-1.70573	L
C	0	-9.30186	2.66267	-1.60129	L
C	0	-8.03311	4.78624	-1.60143	L
C	0	-6.75862	6.85656	-1.94802	L
C	0	-5.45577	8.89429	-2.3459	L
C	0	-4.28465	9.52567	-2.31666	L
C	0	-1.85633	9.47028	-1.92629	L
C	0	0.61904	9.37442	-1.80362	L
C	0	3.08271	9.33702	-1.72651	L
C	0	5.5772	9.30213	-1.71806	L
C	0	6.69587	8.58515	-1.58295	L
H	0	7.64259	9.05885	-1.7303	L
H	0	8.7741	6.93306	-1.40715	L
H	0	9.92112	4.79646	-1.24772	L
H	0	11.08791	2.64189	-1.22995	L
H	0	12.22379	0.50018	-1.35974	L
H	0	10.76583	-4.0072	-1.24968	L
H	0	9.51164	-6.04737	-1.13236	L
H	0	8.24493	-8.13602	-1.33049	L
H	0	4.86124	-11.3475	-1.55483	L
H	0	2.45602	-11.32587	-1.12316	L
H	0	0.03985	-11.29261	-0.83605	L
H	0	-2.40176	-11.22513	-0.96609	L
H	0	-4.82993	-11.20834	-1.16585	L
H	0	-8.10492	-7.77929	-1.29982	L
H	0	-9.24177	-5.63102	-1.37835	L
H	0	-10.38928	-3.46548	-1.54477	L
H	0	-11.53209	-1.34512	-1.78379	L
H	0	-10.18203	3.2044	-1.86179	L
H	0	-8.93445	5.29018	-1.89285	L
H	0	-7.64658	7.37959	-2.23657	L
H	0	-4.21574	10.55415	-2.61528	L
H	0	-1.77429	10.49107	-2.24567	L
H	0	0.65583	10.38874	-2.15122	L
H	0	3.09648	10.35986	-2.02736	L
C	0	12.53156	-2.06365	-1.46423	L
O	0	12.69306	-3.25804	-1.54933	L
O	0	13.57869	-1.21787	-1.64275	L
H	0	14.39572	-1.70301	-1.82672	L
C	0	5.71044	10.73388	-2.06976	L
O	0	4.8218	11.53111	-2.2542	L
O	0	7.00823	11.11899	-2.18093	L
H	0	7.07816	12.0566	-2.41065	L

C	0	-7.22779	-10.2704	-1.3727	L
O	0	-8.35665	-9.85276	-1.48035	L
O	0	-6.97944	-11.6018	-1.47275	L
H	0	-7.79387	-12.10064	-1.62943	L
C	0	-11.89257	1.20441	-1.98883	L
O	0	-12.08438	2.39049	-2.11531	L
O	0	-12.92319	0.32896	-2.11062	L
H	0	-13.75507	0.78783	-2.2956	L
O	0	7.2796	-10.23167	-1.69727	L
H	0	7.21746	-11.17397	-1.89407	L
O	0	-6.64104	9.4633	-2.7287	L
H	0	-6.56397	10.3952	-2.96585	L
O	0	-0.73455	-1.31096	1.53623	H
O	0	-2.4043	2.2602	1.23888	H
H	0	-2.79956	3.06051	1.62196	H
O	0	1.72845	-1.04915	2.29035	H
H	0	0.87575	-0.78666	2.67076	H
C	0	3.79675	-5.41965	-0.04622	H
C	0	6.61377	-4.26129	1.74873	H
O	0	7.37858	-3.58594	2.37819	H
O	0	5.79765	-5.17315	2.2841	H
H	0	5.91082	-5.12957	3.24621	H
C	0	-5.72636	3.54552	0.99983	H
O	0	-5.16551	4.46192	1.54286	H
O	0	-6.24677	2.49536	1.61597	H
H	0	-5.88803	2.49268	2.53175	H
O	0	0.09804	-7.83169	1.77209	H
H	0	0.16745	-8.79089	1.87822	H
O	0	-2.14999	7.22268	0.67845	H
H	0	-2.13147	8.18827	0.73681	H
O	0	0.97153	3.12401	1.46035	H
O	0	4.0998	0.55679	2.04181	H
H	0	3.36103	0.06696	2.44479	H
O	0	1.56179	-5.48223	2.06758	H
H	0	1.22163	-6.35244	2.33436	H
O	0	-0.95289	0.77545	-1.57618	H
O	0	-3.22956	0.3834	-2.50823	H
H	0	-4.07031	0.02084	-2.81808	H
O	0	2.81604	0.70025	-1.38809	H
H	0	3.66974	0.96073	-1.76486	H
C	0	-2.07167	2.7389	4.24316	H
C	0	-1.88626	4.00185	3.75088	H
C	0	-0.55943	4.63879	3.7403	H
C	0	0.47975	4.1412	4.53703	H
C	0	1.78303	4.59316	4.41066	H
C	0	2.05197	5.60537	3.49053	H
C	0	1.01943	6.15236	2.73197	H
C	0	-0.2826	5.6729	2.84511	H

C	0	-3.33649	2.03364	4.03743	H
N	0	-3.37493	0.74835	4.46477	H
C	0	-4.35374	-0.20886	4.14083	H
C	0	-5.01134	-0.19198	2.90544	H
C	0	-5.93969	-1.19435	2.6647	H
C	0	-6.15198	-2.17675	3.62956	H
C	0	-5.40684	-2.11445	4.80067	H
N	0	-4.52542	-1.14793	5.0651	H
S	0	0.05858	2.89274	5.7259	H
O	0	1.23075	2.08151	5.99844	H
O	0	-0.71774	3.47875	6.79914	H
N	0	-0.99009	1.96828	4.78537	H
O	0	-4.28766	2.5805	3.44589	H
O	0	-2.83393	4.72383	3.15446	H
C	0	-0.28654	1.1257	3.79075	H
H	0	-1.04354	0.57516	3.2273	H
H	0	0.30859	1.72764	3.09357	H
H	0	0.36132	0.43716	4.33653	H
H	0	-3.69394	4.24359	3.15999	H
H	0	-2.68024	0.46829	5.14905	H
H	0	2.563	4.14737	5.01914	H
H	0	3.06756	5.96756	3.36928	H
H	0	1.23038	6.94517	2.01891	H
H	0	-1.07961	6.0741	2.22616	H
H	0	-4.7789	0.56557	2.16504	H
H	0	-6.48097	-1.21525	1.72408	H
H	0	-6.86305	-2.97866	3.47121	H
H	0	-5.51947	-2.87402	5.57015	H

B-2 Cartesian coordinates of the optimized geometry of the Piroxicam-Graphene Oxide adsorption complex (OUTPUT FILE)

Center Number	Atomic Number	Atomic Type	Coordinates (Angstroms) X	Y	Z
1	6	0	-1.530632	-3.350027	0.226534
2	6	0	-0.257822	-2.565294	0.290420
3	6	0	-0.343714	-1.078916	0.307857
4	6	0	-1.511456	-0.442094	0.442715
5	6	0	-2.790763	-1.117306	-0.008160
6	6	0	-2.801450	-2.621065	0.110199
7	6	0	0.842613	-0.329289	-0.171230
8	6	0	-1.609654	1.012514	0.859183
9	6	0	-0.318765	1.794143	0.590082
10	6	0	0.920543	1.103890	0.178589
11	6	0	-0.383937	3.259221	0.391020
12	6	0	-1.601175	3.877192	0.409486
13	6	0	-2.842521	3.125897	0.341522
14	6	0	-2.863882	1.720507	0.404601
15	6	0	-4.052872	1.005714	0.338331
16	6	0	-4.043327	-0.492293	0.617180
17	6	0	-5.274519	-1.204715	0.079334
18	6	0	-5.274420	1.682525	0.175388
19	6	0	-1.463697	-4.729899	-0.277599
20	6	0	-3.955077	-3.294696	-0.090502
21	6	0	-1.668734	5.358109	0.673270
22	6	0	-4.065159	3.811039	0.187851
23	6	0	0.974773	-3.226679	-0.172080
24	6	0	2.229959	-2.409394	-0.119762
25	6	0	2.046651	-1.008138	-0.657695
26	6	0	1.002102	-4.562031	-0.421936
27	6	0	3.441680	-3.092789	-0.679266
28	6	0	3.300316	-0.225676	-1.041735
29	6	0	3.270657	1.213006	-0.573310
30	6	0	2.113958	1.866383	-0.247665
31	6	0	4.596558	-0.937801	-0.712345
32	6	0	4.499416	1.929246	-0.665814
33	6	0	0.846194	3.966181	0.171535
34	6	0	0.780221	5.407098	0.100136
35	6	0	2.059521	3.297868	-0.105396
36	6	0	3.256952	4.023450	-0.345656
37	6	0	-5.261586	3.084654	0.143188
38	6	0	-5.225161	-2.560411	-0.155558

39	6	0	-6.490835	-0.480357	-0.096804
40	6	0	-6.518051	0.929210	-0.019605
41	6	0	-0.411608	6.069095	0.260060
42	6	0	-2.916087	5.991581	0.085222
43	6	0	2.016987	6.126299	-0.168916
44	6	0	4.476794	3.335770	-0.579473
45	6	0	3.231193	5.443219	-0.359913
46	6	0	5.723932	1.231847	-0.817357
47	6	0	5.767106	-0.210152	-0.772106
48	6	0	4.594208	-2.365029	-0.730605
49	6	0	3.432702	-4.522780	-0.839575
50	6	0	2.273008	-5.228807	-0.719140
51	6	0	-0.243709	-5.333585	-0.487812
52	6	0	-3.936528	-4.725246	-0.377670
53	6	0	-2.700348	-5.411130	-0.499178
54	6	0	5.689861	4.066825	-0.771027
55	6	0	4.445318	6.174707	-0.621169
56	6	0	1.997157	7.528750	-0.311347
57	6	0	-0.458851	7.562770	0.362869
58	6	0	-2.894356	7.407562	-0.253993
59	6	0	-5.321311	5.910103	-0.374937
60	6	0	-6.578408	3.830111	0.224715
61	6	0	-7.720005	1.609942	-0.297887
62	6	0	-7.682039	-1.195266	-0.440186
63	6	0	-6.429204	-3.300370	-0.480430
64	6	0	-5.130108	-5.403240	-0.673238
65	6	0	-2.683646	-6.755730	-0.897556
66	6	0	-0.203657	-6.714672	-0.906518
67	6	0	2.327165	-6.738874	-0.732851
68	6	0	4.691394	-5.166828	-1.215474
69	6	0	5.909258	-3.102758	-0.591984
70	6	0	7.045285	-0.889304	-0.965459
71	6	0	6.932142	1.965880	-0.982383
72	6	0	4.418700	7.575255	-0.733567
73	6	0	3.184477	8.246351	-0.617596
74	6	0	0.760239	8.235458	-0.219427
75	6	0	-1.734412	8.139928	-0.212087
76	6	0	-4.139235	8.041550	-0.639369
77	6	0	-5.309113	7.322719	-0.688596
78	6	0	-6.500760	5.224413	-0.346913
79	6	0	-7.747398	3.045537	-0.309619
80	6	0	-8.910471	0.898802	-0.626386
81	6	0	-8.876420	-0.505887	-0.669880
82	6	0	-7.642190	-2.609043	-0.589972

83	6	0	-6.385262	-4.686556	-0.695041
84	6	0	-3.886919	-7.455522	-1.130882
85	6	0	-5.102284	-6.767095	-1.012728
86	6	0	-1.405980	-7.408252	-1.104677
87	6	0	1.023528	-7.374050	-1.155172
88	6	0	8.217696	-0.138021	-1.231575
89	6	0	8.151325	1.294462	-1.200411
90	6	0	6.894494	3.379918	-0.976896
91	6	0	5.658085	5.474943	-0.797789
92	6	0	7.092702	-2.285759	-1.015667
93	6	0	5.857132	-4.473538	-1.208701
94	6	0	4.651789	-6.555617	-1.628046
95	6	0	3.500769	-7.269480	-1.530446
96	6	0	-3.838200	-8.855969	-1.528254
97	6	0	-6.341231	-7.454769	-1.281290
98	6	0	-7.573875	-5.383821	-0.993014
99	6	0	-8.832932	-3.322107	-0.902735
100	6	0	-10.071015	-1.243220	-0.988125
101	6	0	-10.113502	1.619358	-0.944580
102	6	0	-8.890899	3.701749	-0.694018
103	6	0	-7.755642	5.870792	-0.718848
104	6	0	-6.582150	7.977027	-1.010169
105	6	0	-4.146422	9.481940	-0.923717
106	6	0	-1.737109	9.563104	-0.560947
107	6	0	0.707291	9.586914	-0.532678
108	6	0	3.108495	9.648214	-0.827190
109	6	0	5.639140	8.312360	-0.997345
110	6	0	6.854104	6.204250	-1.038414
111	6	0	8.090553	4.118544	-1.194826
112	6	0	9.327222	2.042100	-1.425095
113	6	0	9.428107	-0.801074	-1.541295
114	6	0	8.268428	-2.929856	-1.428327
115	6	0	7.111044	-5.088512	-1.650386
116	6	0	5.908433	-7.165736	-2.092478
117	6	0	3.455070	-8.663683	-1.987813
118	6	0	1.027284	-8.690946	-1.639594
119	6	0	-1.393416	-8.753314	-1.545175
120	6	0	-7.516946	-6.791492	-1.253311
121	6	0	-8.779837	-4.693175	-1.078193
122	6	0	-10.046686	-2.594511	-1.075308
123	6	0	-11.284444	-0.504168	-1.229915
124	6	0	-11.326006	0.832994	-1.222907
125	6	0	-10.068615	2.982871	-0.994504
126	6	0	-8.886642	5.146451	-0.825761

127	6	0	-7.726200	7.281139	-1.021422
128	6	0	-6.549363	9.405474	-1.306156
129	6	0	-5.417190	10.099457	-1.264813
130	6	0	-3.003341	10.183318	-0.873039
131	6	0	-0.574810	10.249757	-0.633971
132	6	0	1.897951	10.286915	-0.814048
133	6	0	4.332702	10.373211	-1.073249
134	6	0	5.526142	9.770433	-1.136382
135	6	0	6.805460	7.624564	-1.122261
136	6	0	8.042724	5.519691	-1.218982
137	6	0	9.286713	3.424822	-1.412483
138	6	0	10.553104	1.350991	-1.691271
139	6	0	10.622531	0.010615	-1.760643
140	6	0	9.414854	-2.191831	-1.660594
141	6	0	8.243734	-4.364094	-1.691462
142	6	0	7.054176	-6.473641	-2.082477
143	6	0	5.837932	-8.563863	-2.509352
144	6	0	4.700105	-9.251708	-2.461503
145	6	0	2.290119	-9.329759	-1.996371
146	6	0	-0.176161	-9.362863	-1.818340
147	6	0	-2.635485	-9.447436	-1.722641
148	6	0	-5.133683	-9.535887	-1.715932
149	6	0	-6.283615	-8.863798	-1.598211
150	1	0	-7.214716	-9.366645	-1.747128
151	1	0	-8.433806	-7.309039	-1.457290
152	1	0	-9.676998	-5.231277	-1.313879
153	1	0	-10.942032	-3.137682	-1.306593
154	1	0	-12.168295	-1.069093	-1.432659
155	1	0	-10.940700	3.534188	-1.257740
156	1	0	-9.807116	5.626468	-1.094972
157	1	0	-8.651182	7.760871	-1.264014
158	1	0	-5.419252	11.149470	-1.482510
159	1	0	-3.003841	11.233695	-1.090892
160	1	0	-0.582773	11.294934	-0.873352
161	1	0	1.848038	11.338674	-1.016848
162	1	0	4.259262	11.431860	-1.197783
163	1	0	7.716636	8.144898	-1.306754
164	1	0	8.947269	6.068515	-1.395988
165	1	0	10.188298	3.980029	-1.584505
166	1	0	11.428442	1.942788	-1.848222
167	1	0	10.306327	-2.698553	-1.946398
168	1	0	9.160620	-4.820712	-2.009249
169	1	0	7.966924	-6.933103	-2.400303
170	1	0	4.677408	-10.274503	-2.783329

171	1	0	2.250135	-10.346816	-2.333777
172	1	0	-0.164361	-10.372377	-2.180020
173	1	0	-2.587625	-10.467495	-2.025439
174	6	0	-12.625045	1.484903	-1.501404
175	8	0	-12.844785	2.678825	-1.553926
176	8	0	-13.617175	0.592418	-1.706903
177	1	0	-14.471726	1.018206	-1.891301
178	6	0	-5.206394	-10.977184	-2.042144
179	8	0	-4.269162	-11.736315	-2.190283
180	8	0	-6.475885	-11.419017	-2.168648
181	1	0	-6.521944	-12.367533	-2.378034
182	6	0	6.720315	10.617343	-1.359598
183	8	0	7.874113	10.241292	-1.420864
184	8	0	6.413034	11.923824	-1.493194
185	1	0	7.198930	12.479848	-1.631201
186	6	0	11.938495	-0.598550	-2.059276
187	8	0	12.188442	-1.784170	-2.145794
188	8	0	12.903783	0.325840	-2.240099
189	1	0	13.770152	-0.070296	-2.436414
190	8	0	-7.778930	9.934698	-1.615889
191	1	0	-7.750599	10.886676	-1.799053
192	8	0	7.037757	-9.072154	-2.944515
193	1	0	6.983532	-10.003407	-3.209605
194	8	0	0.657292	1.369144	1.559278
195	8	0	2.516197	-2.128452	1.267232
196	1	0	3.006303	-2.885003	1.629172
197	8	0	-1.797750	0.994983	2.290683
198	1	0	-0.965377	0.680272	2.680065
199	6	0	-4.064824	5.266860	-0.006032
200	6	0	-6.821588	3.949111	1.772768
201	8	0	-7.544916	3.205197	2.390872
202	8	0	-6.084757	4.903821	2.328771
203	1	0	-6.212993	4.873021	3.293975
204	6	0	5.934803	-3.309515	0.971678
205	8	0	5.463211	-4.286926	1.502754
206	8	0	6.400825	-2.252821	1.617007
207	1	0	6.109366	-2.320357	2.552550
208	8	0	-0.454375	7.861651	1.797480
209	1	0	-0.672413	8.799820	1.911766
210	8	0	2.561168	-7.077631	0.653793
211	1	0	2.459323	-8.037712	0.751474
212	8	0	-0.841757	-3.141500	1.469210
213	8	0	-4.062074	-0.725436	2.020375
214	1	0	-3.333448	-0.208872	2.414345

215	8	0	-1.828578	5.443669	2.121473
216	1	0	-1.581500	6.355404	2.357044
217	8	0	0.994971	-0.703670	-1.550832
218	8	0	3.247087	-0.216657	-2.477440
219	1	0	4.066882	0.177172	-2.813014
220	8	0	-2.759036	-0.801867	-1.410637
221	1	0	-3.598918	-1.085000	-1.808567
222	6	0	2.186475	-2.781923	4.258417
223	6	0	2.032428	-4.032037	3.725625
224	6	0	0.730455	-4.720435	3.714078
225	6	0	-0.319785	-4.284663	4.535641
226	6	0	-1.604201	-4.795502	4.435901
227	6	0	-1.847716	-5.803220	3.503050
228	6	0	-0.808789	-6.283676	2.709394
229	6	0	0.474245	-5.748930	2.806160
230	6	0	3.441175	-2.041816	4.086772
231	7	0	3.477966	-0.783910	4.575583
232	6	0	4.467472	0.177415	4.271986
233	6	0	5.028142	0.245183	2.994315
234	6	0	5.983803	1.224394	2.763621
235	6	0	6.311199	2.108877	3.789143
236	6	0	5.653945	1.976496	5.004813
237	7	0	4.750025	1.022035	5.259883
238	16	0	0.074465	-3.043004	5.727614
239	8	0	-1.120387	-2.276628	6.056605
240	8	0	0.861930	-3.628019	6.806431
241	7	0	1.087349	-2.060666	4.835905
242	8	0	4.398962	-2.557114	3.472257
243	8	0	3.013345	-4.705181	3.116654
244	6	0	0.370935	-1.164364	3.892772
245	1	0	1.125180	-0.601006	3.341482
246	1	0	-0.244649	-1.728730	3.183266
247	1	0	-0.245073	-0.475180	4.470702
248	1	0	3.838632	-4.158514	3.135703
249	1	0	2.748386	-0.501923	5.223200
250	1	0	-2.394534	-4.413426	5.073497
251	1	0	-2.847053	-6.212203	3.402168
252	1	0	-1.000090	-7.069982	1.984449
253	1	0	1.265450	-6.103833	2.152816
254	1	0	4.705140	-0.435561	2.213656
255	1	0	6.454012	1.305456	1.788545
256	1	0	7.046754	2.891713	3.647806
257	1	0	5.862828	2.662620	5.820494
